# Family Violence, Sibling, and Peer Aggression During Adolescence: Associations With Behavioral Health Outcomes

**DOI:** 10.3389/fpsyt.2020.00026

**Published:** 2020-02-11

**Authors:** Katherine M. Ingram, Dorothy L. Espelage, Jordan P. Davis, Gabriel J. Merrin

**Affiliations:** ^1^ School of Education, University of North Carolina at Chapel Hill, Chapel Hill, NC, United States; ^2^ University of Southern California, Suzanne-Dworak Peck School of Social Work, USC Center for Artificial Intelligence in Society, USC Center for Mindfulness Science, USC Institute for Addiction Science, Los Angeles, CA, United States; ^3^ Department of Human Development and Family Studies, Texas Tech University, Lubbock, TX, United States

**Keywords:** bullying, substance (drug) abuse, peer deviance, childhood trauma and adversity, adverse child experiences, aggressive behavior

## Abstract

Bullying and sibling aggression can appear as similar behavior, though the latter is comparatively understudied. Aligned with the Theory of Intergenerational Transmission of Violence, research suggests that exposure to family violence increases an individual's risk for perpetrating violence in their own future relationships. Additionally, Problem Behavior Theory suggests that engaging in one problem behavior (e.g., bullying) increases the likelihood of engaging in other problem behavior (e.g., substance use). In Phase 1, this study of middle school students from the U.S. examined how exposure to family violence predicted membership in latent classes of bullying and sibling aggression perpetration (*N* = 894, sampled from four middle schools). In Phase 2, we used mixture modeling to understand how latent classes of family violence, sibling aggression, and bullying predict future substance use, mental health outcomes, and deviance behavior later in high school. Results yielded four profiles of peer and sibling aggression: *high all*, *high sibling aggression*, *high peer aggression*, and *low all aggression*. Youth who reported witnessing more family violence at home were significantly more likely to fall into the *sibling aggression* only and *high all* classes, compared to the *low all* class. Phase 2 results also yielded four classes: a *high all* class, a *sibling aggression and family violence* class, a *peer aggression* class, and a *low all* class. Individuals in the *high all* class were more likely to experience several unfavorable outcomes (substance use, depression, delinquency) compared to other classes. This study provides evidence for pathways from witnessing violence, to perpetrating aggression across multiple contexts, to developing other deleterious mental and behavioral health outcomes. These findings highlight the negative impact family violence can have on child development, providing support for a cross-contextual approach for programming aimed at developing relationships skills.

## Introduction

Aggressive behavior manifests across contexts and environments throughout adolescence, including school (e.g., bullying peers) and home (e.g., sibling aggression). Bullying estimates vary but, in general, 19.3 to 36.5% of middle and high school students report traditional bullying perpetration, and 10.9 to 15.8% of students perpetrate cyberbullying (1–3). However, sibling conflict is comparatively understudied and often understood as normative (4). Some regard sibling rivalry as a marker for positive social development (5, 6). However, Tippett and Wolke (7) identified a homotypic relationship between sibling agression and school bullying: children who bullied a sibling were also likely to bully peers at school and children who were victimized by a sibling were also more likely to be victimized by peers at school. Most children (about 80%) in the U.S. grow up with at least one sibling, and this relationship is touted as one of the longest and most relevant relationships in an individual's life (8). Prior work suggests that sibling aggression is a highly prevalent form of family violence: between 30 and 80% of youth between ages 3 and 17 report being physically assaulted at least once by a sibling during their lifetime (9, 10). An observational study reported between 7 and 12 violent events occurring between siblings in just 3 h of in-home observations (11). These instances appear to be distinctly different and more harmful than developmentally normative sibling conflict. Similar to bullying among peers, sibling aggression is associated with both short- and long-term problems including maladaptive adjustment in adolescence, increased risk for behavioral problems later in life, and greater frequency of engagement in dating violence (12–15).

Observing family violence is a well-established risk factor for youth engagment in both sibling aggression and bullying at school (16–19). Drawing from Social Learing Theory of Aggression (20), when youth observe caretakers or other relevant models invoke aggression in conflict resolution and achieve a desireable outcome, they thereby learn that this behavior is a useful tool. Others have found aggression to be a pathway to gaining social attention (21). This conceptualization also generalizes across contexts. However, the literature has yet to explore the heterogeneity that exists in peer and sibling aggression homotypicality, the extent to which exposure to parental violence predicts this heterogeneity, and the long-term outcomes associated with profiles of exposure to parental violence, peer, and sibling aggression.

### Youth Aggression Across Home and School Contexts

Bullying is defined as recurring acts of aggression perpetrated by an individual or group that are intended to harm another individual. This aggression occurs across a power gradient and can be physical (e.g., hitting), verbal (e.g., name-calling), relational (e.g., social exclusion), or result in damage to property, and can happen in person or through online media (e.g., text messaging, e-mail, chats; 22–24). Among a 2015 nationally representative sample of high school students (grades 9 through 12), 20.2% reported being targets of bullying on school property in the past year (25). In a recent meta-analysis, among the 80 studies assessing bullying among adolescents, 34.5% were agents of traditional bullying and 36% were targets of traditional bullying (26). Prior work has found at the individual level, students who use alcohol and other drugs, are highly impulsive, demonstrate low levels of empathy, and hold traditional beliefs about masculinity are all at heightened risk for bullying others (27–29). At the relational level, students who face heighted risk are those who are exposed to family violence and hostility, have low parental monitoring, have low social support, and have friends involved in delinquent behavior (29–33). At the community level, risk of becoming a bully is associated with exposure to neighborhood violence (30, 34). However, also at the community level, reporting a strong sense of school belonging appears to mitigate risk in bullying involvement (29, 32).

Despite the substantial research on risk factors associated with bullying, fewer have sought to expand empirical knowledge related to sibling aggression. Unfortunately, unlike bullying, there is little consensus among scholars on how to define or operationalize sibling aggression. For example, prior work has used a variety of terminology to define sibling aggression including violence, abuse, bullying, and rivalry (35). Further, some theorists have noted the need to incorporate concepts of harmful intent or repetition of the behavior into the definition of sibling aggression (36). Informed by the current debate about a precise definition, we adopt a holistic approach to understanding sibling aggression outlined by Wolke, Tippett, and Dantchev (37) which includes “any unwanted aggressive behavior(s) by a sibling that involves an observed or perceived power imbalance and is repeated multiple times or is highly likely to be repeated; [sibling] bullying may inflict harm or distress on the targeted sibling including physical, psychological, or social harm” (p. 918). Reported prevalence rates vary by type of aggression (37). However, studies that assess multiple forms of aggression have found that victims of sibling aggression report more physical and verbal forms of aggression and fewer experiences of relational or emotional forms of aggression (38, 39). Similar to the voluminous literature on bullying, prior work notes that youth who experience more than one type of sibling aggression tend to report greater mental health problems (14). Some sibling conflict is normative, harmless, and can even be useful for learning to resolve interpersonal conflict (5, 40). However, prolonged conflict between siblings can be problematic, and differentially affect internalizing and externalizing problems. The link between sibling conflict and heightened depressive mood, loneliness, and poor self-esteem (14, 41, 42). Similarly, sibling conflict has been associated with externalizing problems (e.g., substance use, fighting) among adolescents (43) and increased conduct problems during early adolescence (44). Early longitudinal studies have also noted that, among female siblings, older sister hostility predicted increases in young sister behavior problems over time (45).

Thus, behavioral characteristics of sibling aggression are phenotypically similar to bullying behavior, with most youth reporting multiple forms of aggression (e.g., physical, verbal, relational, emotional; 46). Further, similarities exist across negative outcomes associated with both peer and sibling aggression (37). Additionally, aggressive behavior rarely occurs in isolation and most studies (with a few exceptions, e.g., 7) to date have investigated predictors and correlates of peer and sibling aggression separately. Of relevance, Problem Behavior Theory suggests that engaging in one problem behavior (e.g., bullying) increases the likelihood of engaging in other problem behavior (47). Robust research indicates that the mechanism results from three interlocking systems: the personality system (e.g., one's values and expectations), the perceived environment system (e.g., parental monitoring, peer approval), and the behavior system (e.g., behavior that elicit reward and punishment). These systems interact to either heighten or decrease risk of engagement in problem behavior. Thus, Problem Behavior Theory may explain co-occurrence of bullying and sibling aggression, as well as other problem behaviors such as substance use, peer delinquency, and delinquent behavior. In support of Problem Behavior theory, a number of studies have found that aggression and mental and behavioral health outcomes often co-occur among adolescents (48–51). For example, in a meta-analysis Ttofi, Farringon, Losel, Crago, and Theodorakis (52) found students who bully are significantly more likely to use substances (compared to students who do not bully). They also found that victims of bullying were not more likely than non-victims to use substances, although this finding should be replicated given the small number of studies examined between victimization and later drug use. Unsurprisingly, several studies, have also documented the co-occurrence between perpetration aggression as an adolescent and experiences of depression (53–55).

### Exposure to Family Violence and Adolescent Aggression

One of the most commonly studied correlates of adolescent maladaptive behavior is exposure to family violence. This can include direct violence exposure such as child abuse, neglect, or emotional abuse as well as indirect forms of violence such as witnessing intimate partner violence. For example, prior work on adolescent exposure to violence has reported that 32% witness family assault, 25% witness partner assault, and almost 58% witness assaults in the community (e.g., seeing someone get attacked, hearing gun shots; 9). Witnessing parental conflict, being directly victimized in the home, and witnessing violence in the community has a graded dose-response relationship with negative outcomes (56–58). That is, the more parental violence experienced by youth the greater the risk for future social, mental, and physical health problems. Specifically, several studies have found that exposure to parental violence can increase a youth's risk for later violent experiences. For example, exposure to one or more forms of violence, including interparental violence, has been associated with both perpetration of and victimization from physical violence (59) and sexual violence (60). Recently, Davis and colleagues (56) reported on the heterogeneity in exposure to family and community violence and the association with peer aggression and victimization. Results indicated youth exposed to parental violence early in adolescence and those who reported witnessing increasing community violence were more likely to engage in bullying and be victims of peer aggression. Similar findings have been reported for the association between exposure to family violence (and other household or family characteristics) and sibling aggression. For example, Hardy (61) found that experiences of family stress (defined broadly) was associated with higher rates of sibling aggression. Others have found exposure to maternal aggression such as low warmth, overt aggression, relational aggression, and witnessing higher rates of parent to parent violence have been associated with higher rates of sibling aggression (35, 62–64).

A useful theoretical frame for these findings is the theory of intergenerational transmission of violence (65–67), which is rooted in Social Learning Theory (20). This theory posits that witnessing intimate partner violence early in life (e.g., between parents) increases the risk that an individual will later perpetrate violence in their own relationships (66). Several studies offer support for this theory by identifying witnessing of family violence as one of the most robust and important predictors of future relationship violence (i.e., within romantic and other close relationships) among adults and adolescents (68–70). Notably, Ehrensaft and colleagues (71) found that over the course of 20 years, children who witnessed intimate partner violence were more likely to mimic these patterns as perpetrators and also become victims, compared to children who did not witness intimate partner violence. Further, extant research has found that witnessing intimate partner violence is associated with dysregulated affect, a tendency to blame oneself for negative occurrences, and an overall increase in internalizing problems (72, 73). To summarize known consequences, a 2008 meta-analysis of the effects of exposure to domestic violence on internalizing and externalizing problems found medium to large effect sizes for internalizing problems (*d* = 0.48), externalizing problems (*d* = 0.47), and trauma symptomology (*d* = 1.54; 74). Thus, exposure to parent or family violence appears to be highly influential in a child's life and future outcomes. Regarding mechanisms of violence transfer, Social Learning Theory lends a helpful understanding. According to Bandura (20), the home or community acts as a learning space for socialization and interpersonal relationships. Thus, when aggression is modeled in these spaces, children are learning that these strategies are normal or useful (75). Aggressive interpersonal behavior learned intergenerationally also appear to be cognitively mediated such that observing violence can shape one's belief system to accept violence as normal or acceptable (76). This increases the chances that violence will be employed across contexts (76). Thus, if youth witness positive outcomes of violence perpetration (e.g., an expression of jealousy), schemas develop that accept violence as effective means of conflict resolution with partners (71).

### Current Study

Taken together, these somewhat siloed bodies of evidence suggest that witnessing family violence is associated with future violence perpetration, and that this behavior often manifest similarly across close relationships, often perpetrated by the same individuals. However, these literatures lack insight into the heterogeneity that exists in perpetrating peer and sibling aggression, and the role of witnessing and learning from parental violence in this heterogeneity. Additionally, little is known about long-term outcomes associated with profiles of exposure to parental violence, peer, and sibling aggression perpetration. A deeper understanding of these dynamics has potential to inform intervention on a number of levels. Parents, teachers, and school personel interact with students in separate settings and have varying levels of involvement and connection with each other. However, problem behavior that presents similarly in both settings may benefit from a comprehensive intervention approach that addresses the critical role of learned use of violence.

The current study had two phases. Phase 1 assessed heterogeneity in peer and sibling aggression. Specifically, we tested the theory of intergenerational transmission of violence, by using early adolescent exposure to family violence as predictors of emergent profiles of peer and sibling aggression and how these profiles differ by demographics (i.e., sex, race/ethnicity). We expected at least two distinct profiles of peer and sibling aggression to emerge (H1). Further, and in line with the theory of intergenerational transmission of violence, we expected family violence to predict membership in classes that involve more proximal forms of aggression (e.g., sibling) versus primarily peer aggression (H2). In Phase 2, we included exposure to family violence in a mixture model with peer and sibling aggression. This phase allowed us to examine family violence exposure and engagement in peer and sibling aggression in tandem. We utilized emergent profiles to predict behavioral health outcomes such as substance use (tobacco, alcohol, and cannabis), mental health (depression), and delinquency (peer delinquency and delinquent behavior) by emergent class. We predicted that at least two classes of family violence and peer and sibling aggression would emerge (H3). Specifically, we expected at least one class will emerge that includes high family violence exposure and high endorsement of peer and sibling aggression. Finally, we predicted that youth who endorse high aggression (both peer and sibling) as well as those who are exposed to high levels of family violence would have greater substance use, mental health problems, and engagement in greater delinquent behavior (H4).

## Methods

### Participants

Participants were 894 students from four Midwestern middle schools. Surveys were administered at five time points: Spring/Fall 2008 (Waves 1/2), Spring/Fall 2009 (Waves 3/4), and Spring 2012 (Wave 5). Data were collected every semester in middle school in order to capture temporal associations among risk and protective factors of multiple forms of violence as part of a larger study. At baseline the sample was 29.9% White, 52.3% African American, 4.1% Hispanic, and 1.4% Asian or Pacific Islander and 12.3% multiracial or other. The sample was 50.7% assigned a girl at birth and 49.3% assigned a boy at birth. At baseline, students were in 5^th^ (5.7), 6^th^ (54.9), and 7^th^ (39.4%) grade. See **Table 1** for demographic information.

**Table 1:** Descriptive statistics of all study variables.

Variable	Mean (or *n*) and Standard Deviation (or %)
Sex	
Boy	441 (49.3%)
Girl	453 (50.7%)
Sibling Aggression (Waves 3 and 4)	0.24 (0.35)
Bullying Perpetration (Waves 3 and 4)	0.15 (0.29)
Family Aggression (Waves 1 and 2)	0.99 (0.91)
Tobacco (Wave 5)	0.18 (0.69)
Alcohol (Wave 5)	0.28 (0.75)
Cannabis (Wave 5)	0.73 (1.62)
Depression (Wave 5)	1.02 (0.62)
Delinquency (Wave 5)	0.33 (0.44)
Peer Deviance (Wave 5)	0.82 (0.85)

### Procedure

#### Parental Consent

The current study was formally announced in school newsletters, school district newsletters, and e-mails from the principals prior to the Spring of 2008. Upon receiving approval from the institutional review board (IRB) and district school board, a passive consent process was implemented. A consent form containing information about the purpose of the study and information meetings at each school was distributed to the parents or guardians of every student enrolled in the school. Parents or guardians who did not wish to have their child participate in the study were instructed to complete and return the form. Additionally, students assented (or dissented) to participantion *via* a procedure described on the coversheet of the survey. Surveys were identified only with a unique code number assigned to each student so researchers could track a student's responses across multiple time points, but ensure confidentiality.

#### Survey Administration

Students were initially informed about the nature of the study by one of the six trained research assistants, the principal investigator, or another faculty member who administered the survey. Researchers assured students that their participation in the study was entirely voluntary and that they could skip any question or stop participating in the survey at any time if they were uncomfortable completing it.

Surveys were conducted in classrooms ranging from 10 to 25 students. The survey took approximately 40–45 min to complete. Members of the research team ensured confidentiality during the survey administration by spacing students apart such that they could not see each other's answers. The survey was administered and read aloud while students responded individually. Students could ask questions if they did not understand an item. At least one trained counseling psychology doctoral-level psychology student was on site to provide immediate support and direct students to appropriate services if necessary. Students were also provided the research team's contact information to seek more information about the study, and online resources and hotline numbers to address bullying or violence. Also, students were reminded about in-school resources available to them (e.g., guidance counselors).

### Measures

Each participant completed demographic information that included questions about their sex (boy or girl), age, grade, and race/ethnicity. For race, participants were given six options: African American (not Hispanic), Asian, White (not Hispanic), Hispanic, Native American, or Pacific Islander. Students could mark all that applied. Then, students completed questions about a wide range of scales measuring different forms of violence (e.g., peer, family) as well as risk (e.g., substance use) and protective factors (e.g., social support) associated with aggression and violence at each wave.

#### Family Violence

In Phase 1, we combined Waves 1 and 2 (Spring/Fall 2008). In Phase 2, we combined Waves 3 and 4 (Spring/Fall 2009). The Family Conflict and Hostility Scale (77) measured the level of past year perceived violence in the family environment. The scale contains three items from a larger survey, which was designed for the Rochester Youth Development Study. The three items were: “How often is there yelling, quarreling, or arguing in your household?”, “How often do family members lose their temper or blow up for no good reason?”, and “How often are there physical fights in the household, like people hitting, shoving, or throwing things?” Response options range from 0 (*Never*) through 3 (*Always*) on a 4-point Likert-type scale. One additional item was used that assessed family violence “Before you were 9 years old, did you ever see or hear one of your parents or guardians being hit, slapped, punched, shoved, kicked, or otherwise physically hurt by their spouse or partner?” Given that the survey did not assess *current* risk for students' safety the IRB did not require a mandated reporting protocol. Cronbach's alpha coefficient was.73 for Waves 1 and 2.

#### Bullying Perpetration

The nine-item Illinois Bully Scale (78) was used to assess the frequency of bullying perpetration across Waves 3 and 4. For example, students were asked how often in the past 30 days they engaged in each behavior (e.g., teased other students, excluded others from their group of friends, threatened to hit or hurt another student). Response options included 0 (*Never*), 1 (*1 or 2 times*), 2 (*3 or 4 times*), 3 (*5 or 6 times*), and 4 (*7 or more times*) on a 5-point Likert-type scale. Scores were averaged across Waves 3 and 4. The construct validity of this scale has been supported *via* exploratory and confirmatory factor analysis (78). Although a power differential is not assessed with the Illinois Bully Scale, the scale has correlated positively with peer nominations of bullying (78). The scale correlated moderately with the Youth Self-Report Aggression Scale (*r* = .65; 79), suggesting that it was somewhat unique from general aggression. Cronbach's alpha coefficients were.84 at Waves 3 and 4.

#### Sibling Aggression Perpetration

A sibling aggression perpetration scale (in Waves 3 and 4) was created for this study and included five items that assessed aggression between siblings (80). Items were selected from the University of Illinois Bullying Scale in order to parallel that scale. Five items emerged as a scale in factor analysis, which includes the following: I upset my brother or sister for the fun of it; I got into a physical fight with my brother or sister; I started arguments with my brother or sister; I hit back when a sibling hit me first; and I teased my siblings for the fun of it. Students were asked to indicate how often they did these things to a sibling or other children in their family during that last 30 days. Response options included 0 (*Never*), 1 (*1 or 2 times*), 2 (*3 or 4 times*), 3 (*5 or 6 times*), and 4 (*7 or more times*) on a 5-point Likert-type scale. Scores were averaged across Waves 3 and 4, with a Cronbach's alpha coefficient of.80 were found for Waves 3 and 4.

#### Substance Use: Tobacco, Alcohol, Cannabis

Substance use was measured using three single items addressing tobacco use, alcohol use, and cannabis use at Wave 5. Each participant was asked to indicate how many days in the past 30 days, they did the following: (1) smoked cigarettes, (2) drank at least on full drink of alcohol, and (3) smoked marijuana (weed, grass, hash and bud) on a 7-point Likert-type scale ranging from 0 (*0 Days*) to 7 (*20 to 30 Days).*

#### Depressive Symptoms

The Orpinas Modified Depression Scale (81) includes six items that asks adolescents how often they felt or acted in certain ways (e.g., “Did you feel happy”, “Did you feel hopeless about your future”) in the previous 30 days. Response options are 0 (*Never*), 1 *(Not Often*), 2 (*Sometimes*), 3 *(Often*), and 4 *(Almost Always*) on a 5-point Likert-type scale. Greater scores indicate more depressive symptoms. The Modified Depression scale has demonstrated strong construct validity through factor analyses and good internal consistency (.74) when administered to adolescents 10 to 18 years of age (81, 82). Cronbach alpha coefficient was.84 at Wave 5.

#### Self-Reported Delinquency

This 8-item scale is based on Jessor and Jessor's (47) General Deviant Behavior Scale and asks students to report how many items listed on the measure they took part in during the last year. The scale consists of items such as, “Skipped school”, and “Damaged school or other property that did not belong to you.” Response options included 0 (*Never*), 1 (*1 or 2 times*), 2 (*3 to 5 times*),3 (*6 to 9 times*), and 4 (*10 or more times*) on a 5-point Likert-type scale. A Cronbach's alpha coefficient of.67 was found for Wave 5.

#### Peer Deviance

The Friend's Delinquent Behavior-Denver Youth Survey is a 7-item scale (83) that asks participants to report how many of their friends within the last year “Hit or threatened to hit someone,” “Purposely damaged or destroyed property that did not belong to them,” and “Used alcohol”, to name a few. Response options were 0 (*None*), 1 (*Very Few*), 2 (*Some of them*), 3 (*Most of them*), and 4 (*All of them*) on a 5-point Likert-type scale. Cronbach's alpha coefficient was.88 for Wave 5.

### Analytic Plan

The current study consists of two phases to address our research questions. Specifically, in Phase 1 we used Latent Class Analysis (LCA) to examine heterogeneity in peer and sibling aggression during middle school. LCA is a technique that identifies heterogeneity within a sample (or groups) and classifies individuals based on probability of item endorsement. We used dichotomized peer and sibling aggression perpetration items in our LCA such that non-zero scores were given a value of “1.” We fit models ranging from one to six classes and examined fit statistics to determine if adding an additional class improved model fit. To assess model fit, we used decreases in the negative two log likelihood (-2LL), Akaike Information Criteria (AIC), Bayesian Information Criteria (BIC), and the sample size adjusted Bayesian Information Criteria (aBIC). Further, we utilized non-significant Vuong-Lo-Mendell-Rubin Likelihood Ratio Test (VLRT), the Lo-Mendell-Rubin adjusted likelihood ratio test (LRT), and the bootstrapped likelihood ratio test (BLRT) to indicate that a *k* – 1 class solution is a better fit to the data. Once the best fitting model was established for peer and sibling aggression perpetration, we used early middle school values of family violence to predict class membership, addressing hypotheses of intergenerational transmission of violence. That is, we used family violence as a predictor of emergent peer and sibling aggression perpetration classes *via* multi-nominal logistic regression. We also examined race and gender as predictors of class membership following class enumeration.

After examining how family violence is associated with the sibling and peer aggression classes, Phase 2 included family violence in the latent class model with sibling and peer aggression. That is, we sought to understand how the addition of family violence may influence peer and sibling aggression perpetration class structure. Once the best fitting model was established (following procedures outlined above) we examined distal outcomes across emergent classes using a Wald chi-square test, yielding pairwise comparisons of all class means on each outcome. Specifically, we assessed mean differences between classes across substance use (i.e., tobacco, alcohol, cannabis), mental health (i.e., depression), and deviance (i.e., delinquency, peer deviance) outcomes in high school (Wave 5).

All analyses were conducted using *Mplus* version 8. Missing data ranged from 4–25% on variables across the study period. We used the full information maximum likelihood (FIML) estimator in *Mplus* 8 (84). Missing data patterns were assessed using Little's test of Missing Completely at Random, χ^2^ tests and Pearson's correlations in SPSS (IBM version 26).

## Results

### Descriptive Statistics

**Table 1** presents the means (or *n*) and standard deviations (or *%*) for all predictor and outcomes variables. Little's test of Missing Completely at Random suggested that data were not missing completely at random for H1-H2 [χ ^2^ (367) = 550.48, *p* < .001] nor for H3-H4 {tobacco use outcome [χ ^2^ (438) = 1071.60, *p* < .001]; alcohol use outcome [χ ^2^ (438) = 1089.08, *p* < .001]; marijuana use outcome [χ ^2^ (454) = 1121.06, *p* < .001]; depression outcome [χ ^2^ (421) = 1059.89, *p* < .001]; delinquency outcome [χ ^2^ (438) = 1071.68, *p* < .001]; peer deviance outcome [χ ^2^ (438) = 1091.17, *p* < .001]}. Looking at patterns of missingness among repondents, Asian students were somewhat more likely to have missing data on several items compared to students of other racial identities: tobacco use (χ ^2^ = 7.75, *p = .*006), alcohol use (χ ^2^ = 7.68, *p = .*006), marijuana use (χ ^2^ = 9.35, *p = .*002), delinquency (χ ^2^ = 8.19, *p = .*005), and peer deviance (χ ^2^ = 11.34, *p = .*001). Also, age at baseline was negatively correlated with providing data for the Wave 5 measures including alcohol use, tobacco use, marijuana use, peer deviance, delinquency, and depression (*r* values range between.07 and.17, *p* < .01). Participant sex and other racial identities had no significant associations with providing data.

### H1: Latent Classes of Peer and Sibling Aggression

**Figure 1:**
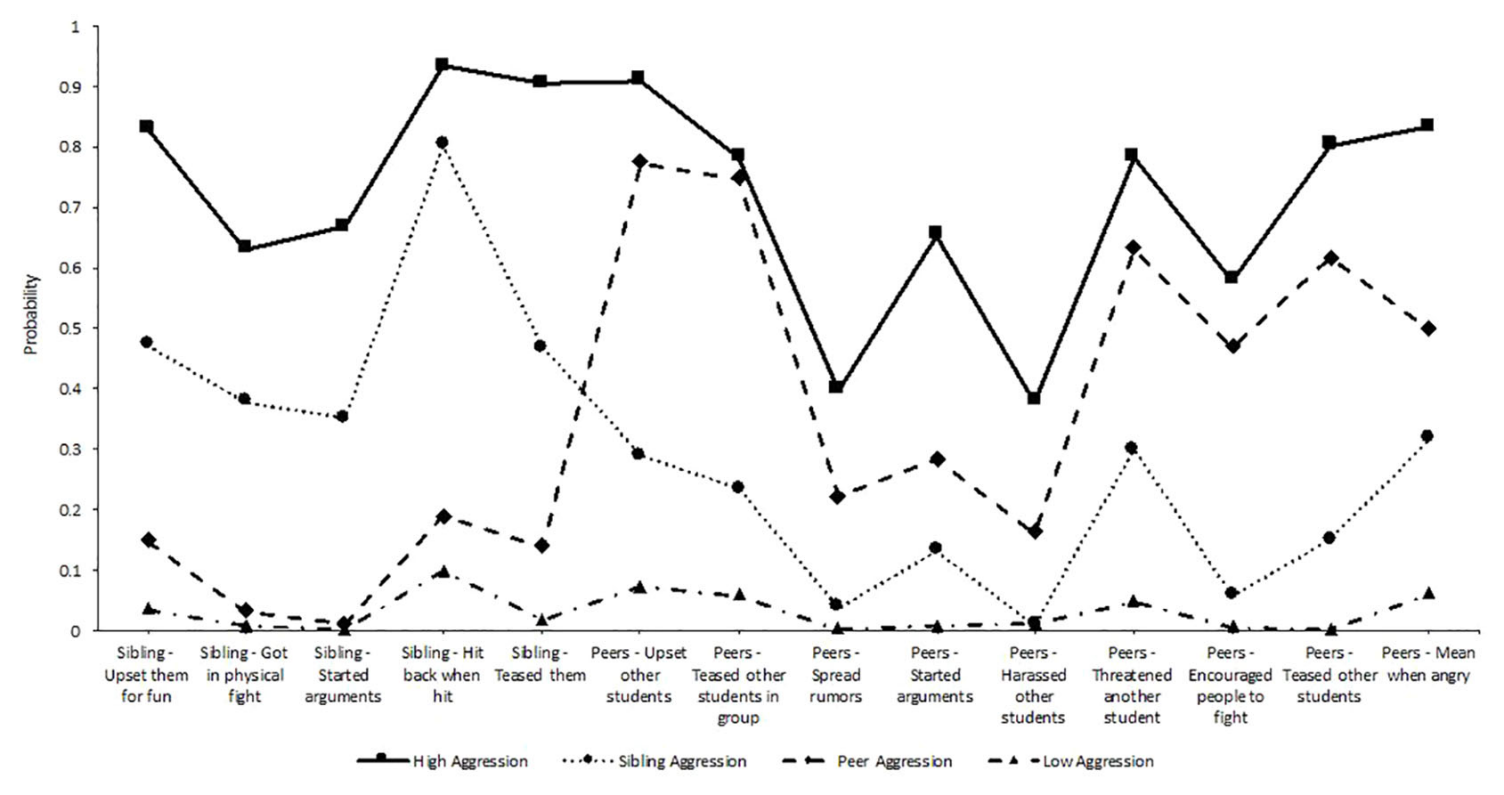
Latent class probabilities for a 4 class solution with sibling and peer aggression (waves 3 and 4).

Phase 1 of the analysis identified classes of peer and sibling aggression during middle school and examined family violence as a predictor of the classes. Results of the LCA with peer and sibling aggression perpetration indicated that a four-class model fit the data best (see **Table 2**). The four-class model had the lowest -2LL, AIC, BIC, CAIC, and AWE among all the models with significant LMRT. The significant LMRT and Adjusted LMRT indicated that the improvement in fit (i.e., reduction in -2LL) from the three to four-class model was statistically significant. An entropy value of 0.85 indicated good class separation (85). The four latent classes included a *high all* aggression class (17.34%, *n* = 155: 83 boys, 72 girls), that had the highest probabilities across both sibling and peer aggression, a *sibling aggression* class (34.56%, *n* = 309: 123 boys, 186 girls, that had high probabilities of engaging in sibling aggression, but low engagement in peer aggression, a *peer aggression* class (8.84%, *n* = 79: 46 boy, 33 girl), that had high probabilities of engaging in peer aggression but low engagement in sibling aggression, and a *low aggression* class (39.26%, *n* = 351: 189 boy, 162 girl), that had the lowest probabilities of engaging in any form of sibling or peer aggression. **Figure 1** displays the probability plot of the four sibling and peer aggression classes. We also looked at demographic predictors (e.g., sex, race/ethnicity) of class membership. Regarding sex assigned at birth, girls were significantly more likely to be in the *sibling aggression* compared to all other classes, with all *p*-values lower than 0.005. Asian students were significantly more likely to fall into the *sibling aggression* class than all other classes. African-American/Black students were most likely to fall into the *high all* and *sibling aggression* classes, though were also significantly more likely to be *in low aggression* than the *peer aggression* class. Latinx students were most likely to fall into *peer agression* and *sibling aggression* classes. White students were most likely to fall into *high all* and *sibling agression* classes. Multiracial students were most likely to be in the *low all* class.

**Table 2:** Model fit Indices for 1 through 5 latent class models with sibling and peer aggression only.

No. of classes	-2LL	AIC	BIC	CAIC	AWE	LMRT *p value*	Adj LMRT *p value*	Entropy
1	13691.35	13719.35	13786.49	13800.49	13923.63	–	–	1.00
2	11313.79	11371.79	11510.86	11539.86	11794.94	.001	.001	.875
3	10835.87	10923.87	11134.89	11178.89	11565.90	.001	.001	.829
**4**	**10565.80**	**10683.80**	**10966.75**	**11025.75**	**11544.70**	**.001**	**.001**	**.850**
5	10454.69	10602.69	10957.57	11031.57	11682.45	.107	.110	.863

-2LL, Negative 2 log likelihood; AIC, Akaike Information Criteria; BIC, Bayesian Information Criteria; CAIC, Consistent Akaike Information Criteria; AWE, Approximate Weight of Evidence Criterion; LMRT, Lo-Mendell-Rubin Test; Adj LMRT, Adjusted Lo-Mendell-Rubin Test. Bolded text indicates solution retained.

### H2: Intergenerational Transmission of Violence: Family Violence Predicting Peer and Sibling Aggression Classes

After identifying the sibling and peer aggression classes, we examined the extent to which family violence was associated with differences between the aggression classes. **Table 3** presents the odds ratios and confidence intervals for a multinomial logistic regression that examines family violence as a predictor of the sibling and peer aggression classes. The results indicated that increases in family violence were associated with higher odds of being in the *high all* [AOR = 2.55, CI = (1.95, 3.33)] and *sibling aggression* [AOR = 1.82, CI = (1.41, 2.35)] classes compared to the *Low aggression* class. In addition, increases in family violence were associated with lower odds of being in the *sibling aggression* [AOR = 0.71, CI = (0.57, 0.90)] and *peer aggression* [AOR = 0.47, CI = (0.32, 0.71)] classes compared to the *high all* class. This indicates that youth who endorse higher rates of family violence are more likely to be in the *high all* (e.g., high endorsement of both sibling and peer aggression) compared to classes that represent sibling or peer aggression only. Finally, youth endorsing higher family violence had lower odds of being in the *peer aggression* [AOR = 0.66, CI = (0.45, 0.98)] class compared to the *sibling aggression* class, indicating witnessing family violence is associated with higher odds of being in a class of individuals that perpetrates aggression towards siblings compared to peers only.

**Table 3:** Class counts for a 4 class solution with sibling and peer aggression.

Classes	Frequency	Percentage
High Aggression	155	17.34%
Sibling Aggression	309	34.56%
Peer Aggression	79	8.84%
Low Aggression	351	39.26%

N = 894.

### H3: Latent Classes of Peer Aggression, Sibling Aggression, and Family Violence

**Figure 2:**
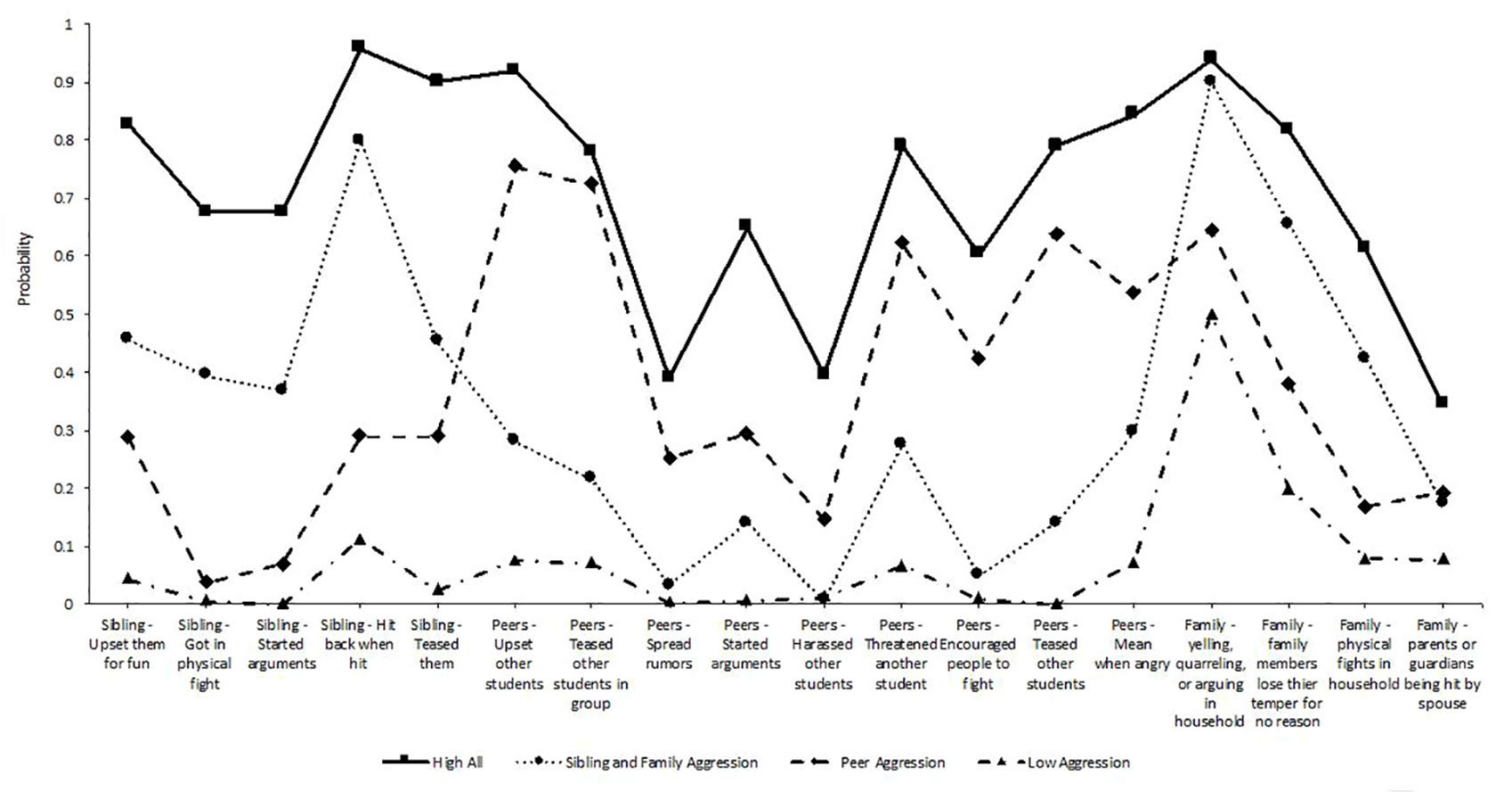
Latent class probabilities for a 4 class solution with sibling, peer, and family aggression (waves 3 and 4).

Phase 2 of the current study identified middle school classes of peer aggression, sibling aggression, and family violence. Further, we examined how emergent classes predicted substance use (tobacco, alcohol, and cannabis), mental health (depression), and deviance (delinquency and peer delinquency) outcomes in high school. Results of the LCA indicated that a four-class model fit the data best (see **Table 4**). The four-class model had the lowest -2LL, AIC, BIC, CAIC, and AWE among all the models with significant LMRT. Like the first LCA, the significant LMRT and Adjusted LMRT indicated that the improvement in fit (i.e., reduction in -2LL) from the three to four-class model was statistically significant. An entropy value of 0.85 indicated good class separation. The four latent classes included a *high all* class (16.56%, *n* = 148: 76 boy, 72 girl), that had the highest probability of endorsing sibling aggression, peer aggression, and witnessing family violence. Further, a *sibling aggression and family violence* class emerged (32.10%, *n* = 287: 119 boy, 168 girl, that had high probabilities of engaging in sibling aggression and witnessing family violence, but low engagement in peer aggression. The third class was labeled *peer aggression* (11.00%, *n* = 98: 55 boy, 43 girl), which had high probabilities of engaging in peer aggression but low engagement in sibling aggression and low exposure to family violence. Finally, a *low aggression* class emerged (40.38%, *n* = 361: 191 boy, 170 girl), that had the lowest probabilities of engaging in any form of sibling or peer aggression as well as low endorsement of family violence. **Figure 2** displays a plot of the four sibling, family, and peer aggression classes.

**Table 4:** Family aggression (waves 1 and 2) predicting sibling and peer aggression classes (waves 3 and 4).

Variables	Family Aggression Predictor	
	OR	95% OR Confidence Interval
**Reference Low All**		
Vs. high all	2.55	[1.95, 3.33]
Vs. sibling aggression	1.82	[1.41, 2.35]
Vs. peer aggression	1.21	[0.81, 1.80]
**Reference High All**		
Vs. sibling aggression	0.71	[0.57, 0.90]
Vs. peer aggression	0.47	[0.32, 0.71]
**Reference Sibling Aggression**		
Vs. peer aggression	0.66	[0.45, 0.98]

Confidence Intervals that include 1 are not significant at p < .05.

We also looked at demographic predictors of class membership. Regarding sex assigned at birth, girls were significantly more likely to be in the *sibling aggression and family violence* class compared to all other classes, with all *p*-values lower than 0.05. Individuals who identify as African-American/Black, White, or another racial identity were no more likely to fall into any one class compared to any other (all *p*-values greater than 0.05). Asian/Pacific Islander as well as Latinx students were equally likely to be in the *high all, sibling and family aggression,* and *peer aggression,* classes and significantly more likely to fall into each compared to the *low all* class.

### H4: Substance Use, Mental Health, and Deviance Outcomes

After identifying the sibling, family, and peer aggression classes, we examined the extent to which the middle school classes predicted differences in substance use, mental health, and deviance in high school. **Table 5** presents the means and standard errors of each outcome variable across each of the four emergent classes. Regarding substance use behavior, the *high all* and *sibling aggression and family violence* classes reported significantly higher rates of alcohol use compared to the *low all* class. In addition, the *high all* and *peer aggression* classes reported higher rates of cannabis use compared to the *sibling aggression and family violence* and *low all* classes. There were no differences found for tobacco use. For the mental health outcome, the *high all* class reported higher levels of depression compared to both the *peer aggression* and *low all* classes. Further, the *sibling aggression and family violence* class reported higher average levels of depression compared to the *low all* class. Considering deviancy, the *high all* class reported higher levels of delinquency compared to *sibling aggression and family violence* class and the *low all* aggression classes. Further, and the *peer aggression* class reported higher average levels of delinquency compared to the *low all* aggression class. There were no class differences found for peer delinquency or tobacco use.

**Table 5:** Model fit indices for 1 through 5 latent class models with sibling, peer, and family aggression.

No. of Classes	-2LL	AIC	BIC	CAIC	AWE	LMRT *p value*	Adj LMRT *p value*	Entropy
1	17726.772	17762.772	17849.0947	17867.0947	18025.41741	–	–	1.00
2	15060.164	15134.164	15311.60511	15348.60511	15674.04623	.001	.001	.870
3	14447.094	14559.094	14827.65352	14883.65352	15376.21305	.001	.001	.837
**4**	**14138.854**	**14288.854**	**14648.53193**	**14723.53193**	**15383.20987**	**.001**	**.001**	**.850**
5	13970.64	14158.64	14609.43634	14703.43634	15530.23269	.626	.627	.825

-2LL, Negative 2 log likelihood; AIC, Akaike Information Criteria; BIC, Bayesian Information Criteria; CAIC, Consistent Akaike Information Criteria; AWE, Approximate Weight of Evidence Criterion; LMRT, Lo-Mendell-Rubin Test; Adj LMRT, Adjusted Lo-Mendell-Rubin Test. Bolded text indicates solution retained.

## Discussion

Adolescence is a period of development when important physiological (e.g., puberty, brain development) and psychological (e.g., expectation of emotion regulation) changes occur in conjunction with an increasing desire for autonomy, higher reliance on peers for social context, and a propensity for experimentation with high risk behavior (e.g., substance use, delinquency; 86). Additionally, some youth also experience high rates of family violence (56–58). Prior research has shown that youth who experience some form of violence (e.g., family, community) are at increased risk of engaging in unhealthy behavior to cope with this adversity. For example, youth who are exposed to violence early in life may have a more difficult time managing internalizing symptomology (e.g., depression) and have higher rates of aggressive behavior (56, 57). Throughout the literature aggression has focused on peer-to-peer and family (parental) violence, thereby limiting scholarly understanding of a unique form of violence that often transcends both spheres (87). Further, minimal research exists on how sibling aggression relates to behavioral health outcomes among youth. In the current study, we explored heterogeneity in peer and sibling aggression, and how exposure to family violence relates to engagement in multiple ecologies of aggression. Additionally, we examined heterogeneity in family violence as well as heterogeneity in peer and sibling aggression and how these dynamics relate to important behavioral health outcomes.

We found ample heterogeneity in sibling and peer aggression. Specifically, results yielded four profiles of peer and sibling aggression: *high all, high sibling aggression only, high peer aggression only, and low all* aggression. This indicates that, among adolescents, there may be specific subgroups of individuals that engage in poly-perpetration (e.g., within the peer and sibling context) and, simultaneously, there may be groups of youth who are only perpetrators of aggression within a specific context. However, our aim in Phase 1 of our study was to, not only understand if there was heterogeneity in multiple aggression social contexts, but also to test the theory of intergenerational transmission of violence by examining early adolescent exposure to family violence as a predictor of emergent profiles of peer and sibling aggression. Specifically, we predicted that exposure to family violence would predict membership into classes that involve more proximal forms of aggression (e.g., among siblings) rather than more distal forms (e.g., among peers). We found that youth who reported witnessing more family violence at home were 82% more likely to fall into the sibling aggression only class (compared to low all), and 155% more likely to fall into the high all class (compared to low class). These findings largely align with previous research linking witnessing violence in the family to engagement in future aggression within relationships (e.g., peers and siblings; 88). Specifically, social learning theory posits that youth will mimic or model behavior that, from their perspective, appear to provide positive rewards (e.g., conflict resolution; 20). For example, youth who witness violence may see aggression (e.g., fighting, teasing, putting others down) as a way to maintain control in relationships or increase feelings of agency. Further, according to the Cycle of Violence model, fighting and/or violence are often ways to relieve interpersonal tension that has built over time and is followed by a short pleasant “honeymoon” phase thus perpetuating the cycle (89). This is in direct relation to the theory of intergenerational transmission of violence where youth reproduce aggression typologies which they were exposed to early on in life within their own relationships (90). Previous studies have used cross-sectional data and linear regression analyses to identify general co-occurrence among these constructs (7, 37). However, the current analyses extend our understanding of these phenomena in several important ways. First, the current study clarifies temporal associations such that previously witnessing family violence is associated with poly-perpetration (e.g., both peer and sibling aggression). Our study aligns with prior longitudinal work noting that parent behavior (e.g., child maltreatment, intimate partner violence) increases the risk of sibling victimization as well as bullying perpetration (62, 35, 91, 92). Further, prior work has reported the absence of parental warmth (93), and the presence of harsh parenting practices (7) are associated with higher bullying behavior between siblings. The current study also provides insight into common profiles of these aggressive behavior and prevalence of each type. Moreover, it provides an updated understanding of behavior that may appear topographically similar (e.g., peer aggression), but have different roots (e.g., sibling aggression). For example, students that exhibit peer aggression only (no sibling aggression) are less likely to have witnessed family violence than students who exhibit peer and sibling aggression (poly-perpetration). Thus, an understanding of commonly occurring profiles and their correlates, especially across domains that do not share informants (i.e., parents see behavior at home, teachers see behavior at school), is critical to intervention policy and programming.

In Phase 2 of our study, we conducted a mixture model using peer aggression, sibling aggression, and family violence items which allowed us to examine classes of common combinations of involvement in each. This analysis yielded four classes: a *high all* class, a *sibling aggression and family violence* class, a *peer aggression* class, and a *low all* class (**Table 6**). We then examined mental and behavioral health outcomes across emergent classes (**Table 7**). It is interesting that our profiles extracted a class that included family violence and sibling aggression in addition to a class that reported high prevalence rates of both aggression typologies and family violence. This allows us to compare, directly, outcomes for aggression classifications that occur in the family with aggressive behavior that occur in the family and peer context. Looking across outcomes, the *high all* aggression class was significantly more likely to experience deleterious outcomes, on average, compared to the *low all* aggression group (specifically looking at depression, substance use, and deviance). Across some outcomes, specifically cannabis use and deviance, the *peer aggression* only class emerged as significantly more likely to experience undesirable outcomes compared to the *low all* aggression group. Further, youth in the *high all* class reported more alcohol use, cannabis use, and delinquency compared to youth in the *sibling aggression and family violence* class, indicating the addition of peer aggression (the only difference between these classifications was endorsement of peer aggression) is a vital component for long term problem behavior. Thus, in general, youth who engage in multiple forms of aggressive behavior and are exposed to high levels of family violence are more likely to engage in problematic behavior over and above single aggression typologies (e.g., peer and sibling only). Our results are akin to recent work assessing poly-perpetration. For example, in a longitudinal prospective cohort design, youth who reported abuse or neglect (e.g., child maltreatment) were more likely to be perpetrators of subsequent criminal violence, child abuse, and intimate partner violence (94). Milaniak and Widom (95) also found that the youth with early childhood violence exposure were more likely to be poly-perpetrators of violence compared to youth without such histories. Further, in this sample, gender and racial associations with class membership were not particularly strong. This study may not have sensitively captured relevant cultural dynamics of interpersonal violence, but perhaps these dynamics are found to some degree across racial and gender groups. However, in both analyses, girls were significantly more likely to be in the classes characterized by sibling agression (and not peer aggression). Perhaps these analyses support prior work (45) in identifying older sister aggression as a distinct behavior from general sibling aggression. Alternatively, perhaps for girls, aggression is more likely to occur in the home and is regulated across contexts whereas boys are more commonly socialized to exhibit the same behavior across contexts with less regulation. However, more information about gender and age of aggression targets among siblings is needed to draw inferences [i.e. though we know the participant is a girl, we have no information regarding sex of her sibling(s)].

**Table 6:** Class counts for a 4 class solution with sibling, peer, and family aggression.

Classes	Frequency	Percentage
High Aggression	148	16.56%
Sibling and Family Aggression	287	32.10%
Peer Aggression	98	11.00%
Low Aggression	361	40.38%

N = 894.

**Table 7:** Middle school latent classes (waves 3 and 4) predicting depression, delinquency, peer deviance, and substance use outcomes in high school (wave 5).

Variable	1. High Aggression	2. Sibling and Family Aggression	3. Peer Aggression	4. Low Aggression	Chi-Square Comparisons
	Mean/SE	Mean/SE	Mean/SE	Mean/SE	*p* < .05
Substance Use					
Tobacco	0.37 (0.13)	0.11 (0.04)	0.32 (0.14)	0.12 (0.05)	
Alcohol	0.50 (0.14)	0.33 (0.07)	0.30 (0.10)	0.16 (0.05)	1, 2 > 4
Cannabis	1.18 (0.27)	0.56 (0.13)	1.36 (0.32)	0.50 (0.11)	1, 3 > 2, 4
Mental Health					
Depression	1.26 (0.10)	1.11 (0.06)	1.00 (0.09)	0.88 (0.43)	1 > 3, 4; 2 > 4
Deviance					
Delinquency	0.53 (0.65)	0.33 (0.04)	0.42 (0.06)	0.24 (0.29)	1 > 2, 4; 3 > 4
Peer	0.95 (0.13)	0.83 (0.08)	0.86 (0.12)	0.76 (0.06)	
Delinquency					

Results from the current study offer further support for Problem Behavior Theory as well as Social Learning Theory. That is, our study extends Problem Behavior Theory such that youth who are poly-perpetrators of violence *and* experience high rates of family violence are more likely to experience multiple problem behavior later in life. Further, our results extend Social Learning Theory such that the cycle of violence, which is often discussed in relation to exposure to family violence, is not just limited to the family sphere. Especially for individuals who have experienced family violence, peer aggression must be integrated into our understanding of how youth are perpetuating learned violence. Additionally, the finding that youth exhibiting the highest rates of aggression are also most likely to use substances holds implications for how peers become involved in substance use and deviant acts. Given that peer and sibling bullying inherently occur across a power dynamic, perhaps victims are also coerced either implicitly or explicitly into using substances or engaging in deviant behavior. This dynamic may partially account for individuals in the peer-only or low aggression classes reporting high rates of, for example, cannabis use and delinquency. Finally, while we did find that poly-perpetration in addition to family violence was associated with long-term problem behavior, this class also reported the highest rates of depression. Extant literature has described the entanglements between problem behavior (particularly involving aggression and substance use) and depressive symptomology. Thus, it is vital that schools, practitioners, and researchers continue to explore not just problem behavior among poly-perpetrators of aggression, but also mental health outcomes as these youth may be at most risk of experiencing long term mental health problems. Findings from this study underscore the importance of assessing sibling violence when working with youth exhibiting deleterious behavior in school.

### Limitations and Conclusion

The current study should be interpreted in the context of several limitations. First, the current data were drawn from only one region in the U.S., thus limiting generalizability. Second, we did not capture count of siblings, but instead asked students to think about either siblings or other children in their families when answering the sibling aggression measures. Regarding the classes that report peer aggression only, we cannot distinguish between students in this class who do not have siblings and students who do have siblings but only engage in aggression at school. Further, we did not assess changes in peer and sibling aggression as a function of exposure to family violence. Future research may wish to investigate how exposure to family violence is associated with trajectories of peer and sibling aggression. Third, we did not assess poly-victimization as a precursor to peer and sibling aggressive behavior. Future work should investigate how poly-victimization (e.g., exposure to multiple types of violence such as family, community, and peer) relates to poly-perpetration. Also, power dynamics based on identity components among agents and targets of both sibling and peer aggression were not captured in this study. Future work may consider assessing self-reported gender identity (including non-binary and trans identities), sexuality, disability, and other relevant identities. This may allow for a more nuanced analysis of identity-based agent, target, and aggression profiles. Further, the current study did not measure socio-economic status (SES). Two studies have identified inverse correlations between SES and sibling violence, indicating associations among these constructs (35, 62). Additionally, an item assessing family violence included the word “spouse,” which some students may not have understood. This item also did not ask about other forms of romantic and/or cohabitating relationships among caregivers. Finally, the current study does not provide any causal links from exposure to family violence to peer and sibling aggression. More nuanced methodologies that allow for the disaggregation of within- and between-person variance may provide a closer approximation of causality.

The current results provide evidence for pathways from witnessing violence, to perpetrating aggression across multiple contexts, to developing other deleterious mental and behavioral health outcomes. These findings highlight the deleterious impact of family violence on child development, providing support for a cross-contextual approach for programming aimed at developing relationships skills to prevent conflict and violence. Future directions should consider exploring protective factors that interrupt the pathways from witnessing violence in the family to perpetration aggression with peers, especially in the context of racial or ethno-cultural differences. Additionally, future work should aim to identify other predictive differences (e.g., environmental, personality) between perpetrating sibling only and sibling and peer aggression among students who do witness family violence. In short, the current study extends several theoretical orientations and provides the first account of how exposure to family violence is associated with poly-perpetration of aggression across both sibling and peer context. Further, this study notes long-term problem behavior such as substance use and delinquency as well as mental health correlates of youth who have experienced family violence and perpetrate aggression toward both peers and siblings. Programming that addresses poly-perpetration is vital for prevention of long-term problems, especially among those youth who have experienced greater amounts of family violence.

### Ethical Considerations

Our study included passive consent (e.g., waiver of consent from parents). Parents were mailed two copies of a parent information letter through US mail and through an email from the school. These letters were sent per prior approval from our IRB. In Spring 2012, two copies of a parental information letter were sent to parents of students in participating districts that included a description of the study and an option to deny their son/daughter participation. Student waiver of parental consent was used for several reasons. First, a waiver of parental consent gave us the best chance of obtaining a representative sample and the most accurate information given the nature of this study. Moreover, evidence suggests that active consent procedures can result in biased samples with under-representation of lower achieving and less involved students (96). Our decision to use a waiver of active consent was in line with American Psychologist Association (APA) requirements. As noted by Kobor and Studwell (97), “APA has worked in coalition with a number of science, education, and public health organizations to protect the ability of scientists to conduct research in schools without having an absolute requirement of prior, written parental consent.” As such, although APA states that, at a minimum, parents should be informed about school-based research projects and offered an opportunity to withdraw their children, APA's position maintains that even in the case of sensitive research topics active consent is not a necessity.

## Data Availability Statement

The datasets generated for this study are available on request to the corresponding author.

## Ethics Statement

The studies involving human participants were reviewed and approved by University of Illinois at Urbana Champaign. Written informed consent for participation was not provided by the participants' legal guardians/next of kin because passive consent was used—school based research.

## Author Contributions

KI helped conceive the analyses in this manuscript, draft the manuscript, and addressed revisions. DE wrote the grant that allowed for data collection, collected the data, helped conceive the study and drafted portions of the study. GM helped analyze the data, interpret results, and drafted parts of the manuscript. JD helped conceive the analyses in manuscript, interpreted results, and helped write the manuscript. All authors read and approved the final version of the manuscript.

## Funding

Middle school data in this manuscript were drawn from a grant from the Centers for Disease Control and Prevention (1U01/CE001677) to DE (PI). High school data in this manuscript were drawn from a grant from the National Institute of Justice (Grant #2011-90948-IL-IJ) to DE (PI).

## Conflict of Interest

The authors declare that the research was conducted in the absence of any commercial or financial relationships that could be construed as a potential conflict of interest.
